# Glioblastomas within the Subventricular Zone Are Region-Specific Enriched for Mesenchymal Transition Markers: An Intratumoral Gene Expression Analysis

**DOI:** 10.3390/cancers13153764

**Published:** 2021-07-27

**Authors:** Diana J. Z. Dalemans, Sharon Berendsen, Kaspar Draaisma, Pierre A. Robe, Tom J. Snijders

**Affiliations:** 1 UMC Utrecht Brain Center, Department of Neurology and Neurosurgery, University Medical Center Utrecht, 3584 CX Utrecht, The Netherlands; dalemansdiana@gmail.com (D.J.Z.D.); s.berendsen-2@umcutrecht.nl (S.B.); K.Draaisma@umcutrecht.nl (K.D.); t.j.snijders@umcutrecht.nl (T.J.S.); 2 Department of Neurology, Erasmus University Medical Center Rotterdam, 3015 GD Rotterdam, The Netherlands; 3 Department of Human Genetics, University Hospital Liege, 4000 Liege, Belgium

**Keywords:** glioblastoma, subventricular zone, intratumoral heterogeneity, gene set enrichment analysis, (epithelial-)mesenchymal transition

## Abstract

**Simple Summary:**

Involvement of the subventricular zone (SVZ) in glioblastoma is associated with poor prognosis and is associated with specific tumor-biological characteristics. In this study, we demonstrate that patient-derived glioblastoma samples from within the SVZ region show increased (epithelial-)mesenchymal transition and angiogenesis/hypoxia signaling as compared to glioblastoma samples from the same patient from outside the SVZ. These results suggest that intratumoral alterations in oncogenic signaling could be mediated by the SVZ microenvironment. Our findings offer rationale for specific targeting of the SVZ in the development of glioblastoma therapy.

**Abstract:**

*Background:* Involvement of the subventricular zone (SVZ) in glioblastoma is associated with poor prognosis and is associated with specific tumor-biological characteristics. The SVZ microenvironment can influence gene expression in glioblastoma cells in preclinical models. We aimed to investigate whether the SVZ microenvironment has any influence on intratumoral gene expression patterns in glioblastoma patients. *Methods*: The publicly available Ivy Glioblastoma database contains clinical, radiological and whole exome sequencing data from multiple regions from resected glioblastomas. SVZ involvement of the various tissue samples was evaluated on MRI scans. In tumors that contacted the SVZ, we performed gene expression analyses and gene set enrichment analyses to compare gene (set) expression in tumor regions within the SVZ to tumor regions outside the SVZ. We also compared these samples to glioblastomas that did not contact the SVZ. *Results:* Within glioblastomas that contacted the SVZ, tissue samples within the SVZ showed enrichment of gene sets involved in (epithelial-)mesenchymal transition, NF-κB and STAT3 signaling, angiogenesis and hypoxia, compared to the samples outside of the SVZ region from the same tumors (*p* < 0.05, FDR < 0.25). Comparison of glioblastoma samples within the SVZ region to samples from tumors that did not contact the SVZ yielded similar results. In contrast, we observed no differences when comparing the samples outside of the SVZ from SVZ-contacting glioblastomas with samples from glioblastomas that did not contact the SVZ at all. *Conclusion:* Glioblastoma samples in the SVZ region are enriched for increased (epithelial-)mesenchymal transition and angiogenesis/hypoxia signaling, possibly mediated by the SVZ microenvironment.

## 1. Introduction

Glioblastoma is the most common primary malignant brain tumor. The prognosis is poor, with a median survival of about 15 months, despite intensive treatment including maximal safe resection and temozolomide-based chemoradiation [1].

The subventricular zone (SVZ), lining the walls of the lateral ventricles of the brain, has been of increasing interest in recent glioblastoma research. One key aspect of the SVZ is the neural stem cell niche it harbors. These astrocyte-like neural stem cells are hypothesized as the cells of origin of glioblastoma [2,3,4]. SVZ contact is associated with poor prognosis in glioblastoma patients. The underlying mechanisms of this adverse prognostic effect are possibly mediated by intrinsic tumor-biological characteristics. However, studies that have focused on exploring tumor-biological differences between glioblastomas with and without SVZ contact have shown inconclusive results [5,6,7,8,9,10,11,12]. In all previous gene expression studies on this subject, bulk tumor data were analyzed. Intratumoral heterogeneity, a known characteristic in glioblastoma, was not taken into account [13,14]. For example, it has been shown that multiple subtypes of the Verhaak classifier for glioblastoma (i.e., proneural, neural, classical and mesenchymal) can be found in the same tumor [14]. It is proposed that the tumor microenvironment contributes to this intratumoral heterogeneity [15].

Hence, our study focused on exploring oncogenic signaling in patient glioblastoma samples within the SVZ microenvironment, to explore a possible influence of the SVZ microenvironment on glioblastoma gene expression. We made use of glioblastoma tissue samples from the publicly available Ivy Glioblastoma Atlas, which contains clinical, radiological and whole exome sequencing data from multiple regions from en bloc resected glioblastomas [16]. In the tumors that contacted the SVZ, we performed gene expression analyses and gene set enrichment analyses to compare gene (set) expression in tumor regions within the SVZ to tumor regions outside the SVZ, within the same tumors. We also compared these samples to glioblastomas that made no contact with the SVZ.

We hypothesized that gene expression in glioblastoma samples from within the SVZ region show unfavorable oncogenic signaling characteristics, as a possible effect of the SVZ microenvironment.

## 2. Materials and Methods

### 2.1. Patient Cohort and Baseline Characteristics

Patients were selected from the Ivy Glioblastoma Atlas, an online accessible database that contains clinical, genomic and histologic information on patients with glioblastoma (http://glioblastoma.alleninstitute.org/ accessed on 15 March 2019) [16]. Adult patients with a primary IDH1-wildtype glioblastoma were included. Subsequently, clinical features including gender, age, initial KPS, extent of resection, adjuvant radiotherapy and/or chemotherapy and survival were collected. Additionally, molecular characteristics including EGFR amplification, MGMT methylation, PTEN status and molecular subtype (classical, mesenchymal, proneural and neural) [13] were collected. The baseline characteristics were compared between patients with SVZ-contacting glioblastoma and patients with glioblastoma without SVZ contact. Survival of patients with SVZ-contacting glioblastoma and patients with glioblastoma without SVZ involvement was compared with Kaplan-Meier curves and a log rank test.

### 2.2. MRI Analysis and Tissue Block Selection

SVZ involvement in glioblastomas was inspected on pre-operative T1-weighted post-contrast MRI images. SVZ contact was defined as contact of contrast-enhancing tumor with a lateral ventricle. The presence of SVZ contact was evaluated independently by two investigators (D.J.Z.D. and S.B.), with blinding to the clinical and genomic data. In case of disagreement, a third investigator (T.J.S.) gave the conclusive statement.

Along with the MRI scans, macroscopic images of en bloc resected tumors were available. The spatial orientation of the resected tumor was provided (i.e., anterior, posterior, lateral and medial side). The tumor tissue had been divided subsequently into multiple tissue blocks, for in situ hybridization and RNA expression analysis, as described in the original paper [16]. In this way, tissue blocks could be matched to their anatomical location on the MRI images, and tissue blocks from the SVZ region were selected.

These selection processes resulted in three groups of samples for further analysis and comparison: (a) glioblastoma samples within the SVZ (*withinSVZ*-samples), (b) samples outside of the SVZ from the same SVZ-contacting glioblastomas (*outsideSVZ*-samples), and (c) samples from tumors without any SVZ contact (*noSVZcontact*-samples).

### 2.3. RNA Sequencing Data Selection

Sampling of tissue blocks for RNA sequencing is described in the original paper [16] and was based on anatomical structural features that are commonly seen by neuropathologists in glioblastoma tissue sections stained with hematoxylin and eosin (H&E). The major structural regions were Leading Edge, Cellular Tumor and Infiltrating Tumor. Leading Edge was defined as the outermost boundary of the tumor, where the ratio of tumor to normal cells is about 1–3/100. Cellular Tumor constitutes the major part of core, where the ratio of tumor to normal cells is about 100/1 to 500/1. Infiltrating Tumor is the intermediate zone between the Leading Edge and Cellular Tumor, where the ratio of tumor cells to normal cells is about 10–20/100 [16]. In our study, only RNA sequencing data sampled by Cellular Tumor were included, as this yields the most tumor-specific data.

### 2.4. RNA Sequencing Data Analysis

Detailed information on tissue processing, RNA isolation, sequencing, quality control and alignment was described previously [16]. Subsequent RNA sequencing analysis was performed with aligned files in bam format with R (version 3.5.2). Count matrices were generated with the GenomicAlignments package (version 1.18.1). Genes with low read counts were dropped and TMM normalization for library size was performed to eliminate composition biases between libraries (edgeR package, version 3.24.3). We performed differential expression analyses by fitting a linear mixed model, with the location of the sample as fixed effect and patient ID as random effect, with the dream (differential expression analysis for repeated measures) analysis from the variancePartition package (version 1.12.3). We used mixed models, as this analysis method allows for correction of gene expression correlations in tumor samples from the same patient. Genes with a p-value adjusted for multiple testing with Benjamini-Hochberg’s False Discovery Rate (FDR) below 0.05 were considered to be significantly differentially expressed. A heatmap was constructed to visualize the most differentially expressed genes across tumor locations (gplots package).

Subsequently, we performed Quantitative Set Analysis for Gene Expression (qusage package) in order to identify differential enrichment of gene sets between the groups. The ggen function of the qusage package (version 2.16.1) allowed us to incorporate the linear mixed model with the location of the tissue sample as fixed effect and patient ID as random effect. This analysis was performed with the hallmark gene sets [17] from the Molecular Signatures Database. Enriched gene sets with a *p* < 0.05 and a false discovery rate (FDR) <0.25 were considered significant. The results were visualized with pathway enrichment plots (qusage package).

## 3. Results

**Figure 1:**
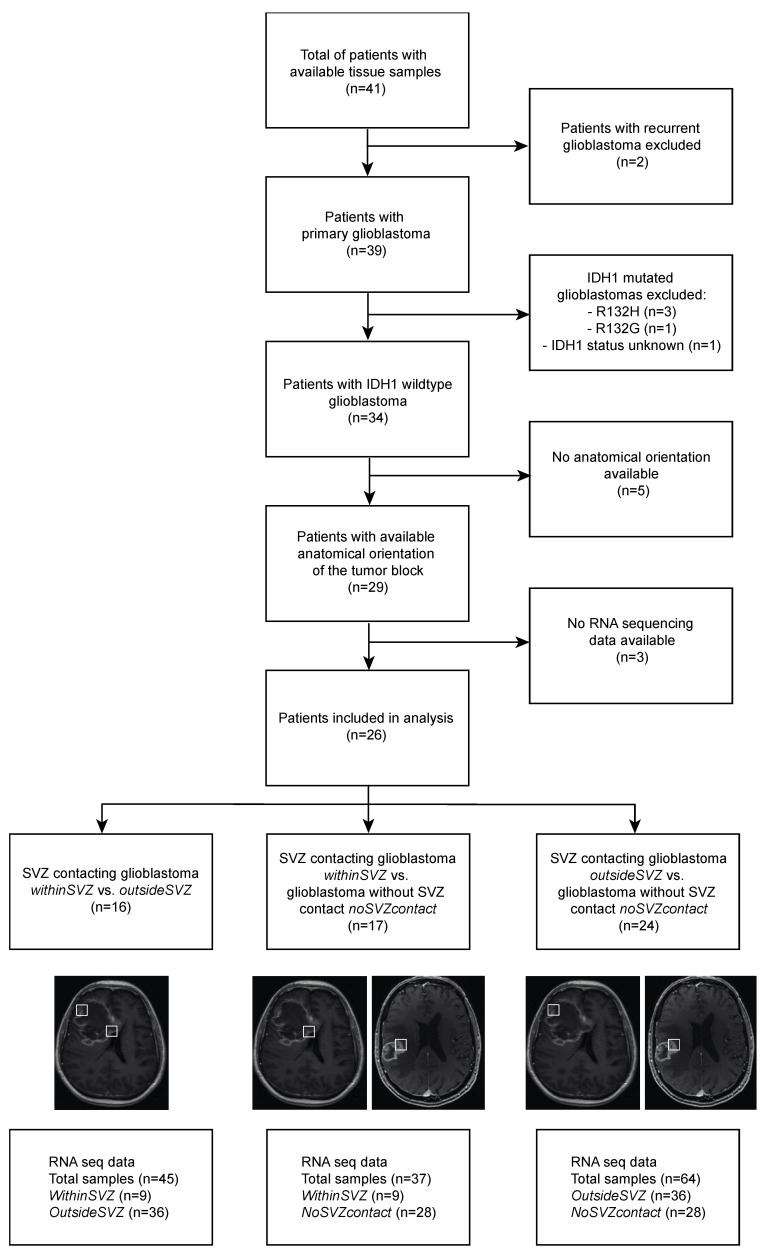
Flowchart.

**Figure 2:**
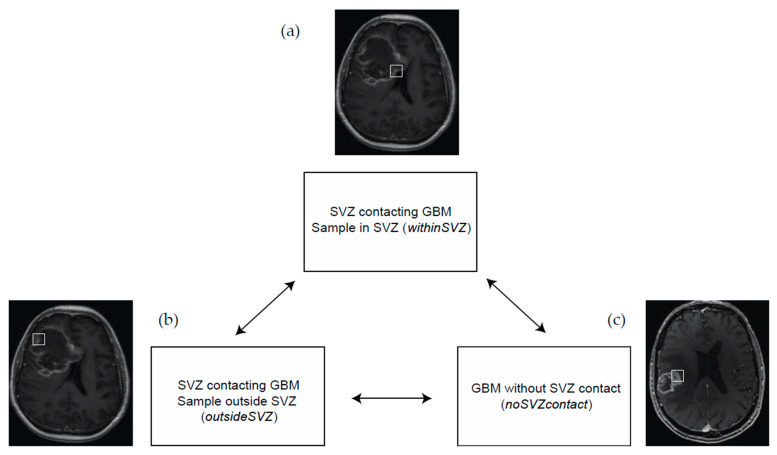
Analysis overview. In our study, we compare gene (set) expression between (**a**) glioblastoma samples within the SVZ (*withinSVZ* samples), (**b**) samples outside of the SVZ from SVZ-contacting glioblastomas (*outsideSVZ* samples), and (**c**) samples from tumors without SVZ contact (*noSVZcontact* samples).

The Ivy Glioblastoma Atlas contains a total of 41 glioblastoma patients, of whom 34 had IDH1 wildtype primary glioblastoma (Figure 1). Due to absence of spatial orientation data of the tumor or absence of RNA sequencing data, eight patients were excluded (Figure 1). As a result, a total of 26 patients were included in our analysis (Figure 2).

Patients with SVZ-contacting glioblastoma did not differ significantly from patients with glioblastoma without SVZ contact with respect to age, initial KPS, extent of resection, treatment, PTEN status, MGMT methylation status, EGFR amplification status and EGFRvIII mutation status (Table 1). Median overall survival was shorter in patients with SVZ-contacting glioblastoma (442 days vs. 544 days in patients with glioblastoma without SVZ contact), but this observation did not reach a statistically significant difference. (Table 1, Supplementary Figure S1). Finally, molecular tumor subtype(s) were comparable in SVZ-contacting glioblastoma and glioblastoma without SVZ contact. Often, more than one subtype was found in the same tumor (Supplementary Table S1).

### 3.1. Differential Enrichment of Oncogenic Gene Sets across Tumor Regions: Intratumoral Analysis

First, we analyzed RNA expression in all tumors with SVZ contact. Within the same tumors, we compared the *withinSVZ*-samples to the *outsideSVZ*-samples. In this analysis, 45 tumor samples (nine *withinSVZ*-samples and 36 *outsideSVZ*-samples) were included, from sixteen patients (Figure 1).

**Figure 3:**
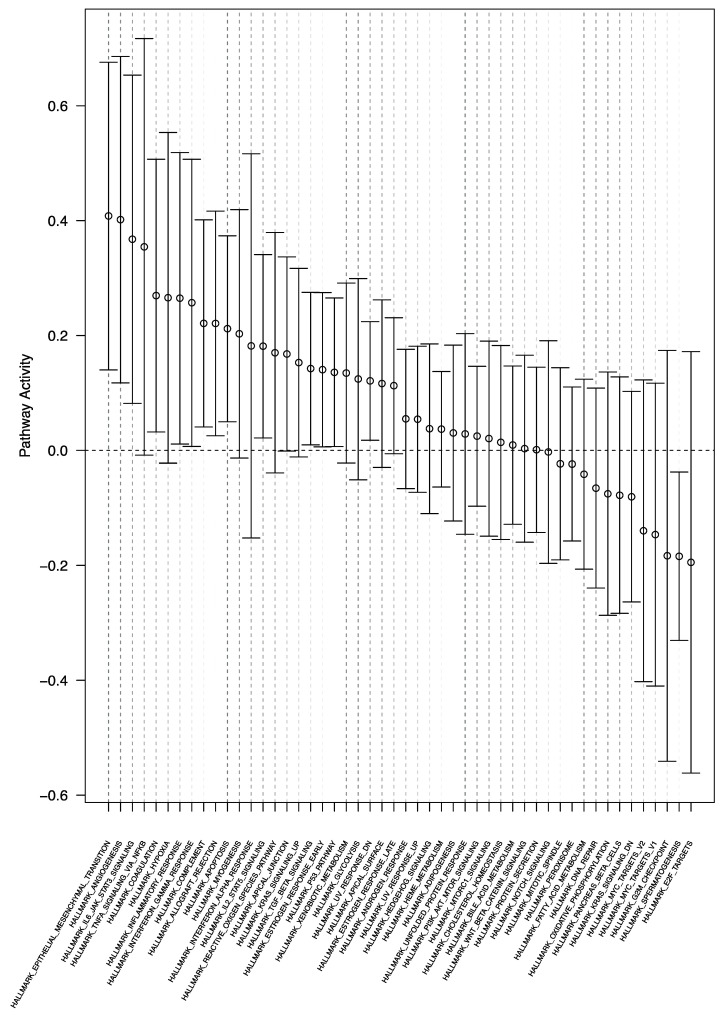
Pathway enrichment plot of the within SVZ contacting glioblastoma (*withinSVZ-*samples vs. *outsideSVZ-*samples) analysis. Gene set enrichment analysis of the *withinSVZ-*samples vs. *outsideSVZ*-samples showed differential enrichment of 24 gene sets (*p* < 0.05, FDR < 0.25), which are shown on the *x*-axis. The relative gene set activity (log fold change) is displayed on the *y*-axis. The 95% confidence intervals are represented by the horizontal bars. Most gene sets were enriched in the *withinSVZ*-samples group, as shown by their positive log fold changes.

After adjustment for multiple testing, no single gene was significantly differentially expressed between tumor samples from within and outside of the SVZ (Supplementary Figure S2). Gene set expression analysis, however, showed differential expression of 24 gene sets (*p* < 0.05, FDR < 0.25) (Supplementary Table S2, Figure 3). In the *withinSVZ*-samples, we observed the most increased enrichment of gene sets involved in (epithelial-)mesenchymal transition, angiogenesis, and NF-κB and JAK/STAT3 signaling compared to the *outsideSVZ*-samples from the same tumors (Supplementary Table S2).

### 3.2. Differential Enrichment of Oncogenic Gene Sets in Glioblastomas in the SVZ Region: Intertumoral Analysis

In this analysis, 37 tumor samples (nine *withinSVZ*-samples and 28 *noSVZcontact*-samples, Figure 2) from 17 patients were included.

The only significantly differentially expressed gene between the *withinSVZ*-samples and the *noSVZcontact*-samples was DCAF4, which was downregulated in the *withinSVZ*-group (logFC = −1.50, adjusted *p* = 0.002). The separate expression values (log counts per million) of DCAF4 per sample are shown in Supplementary Figure S3. The results for the fifty genes with the lowest (unadjusted) *p*-values are shown in a heatmap (Supplementary Figure S4).

**Figure 4:**
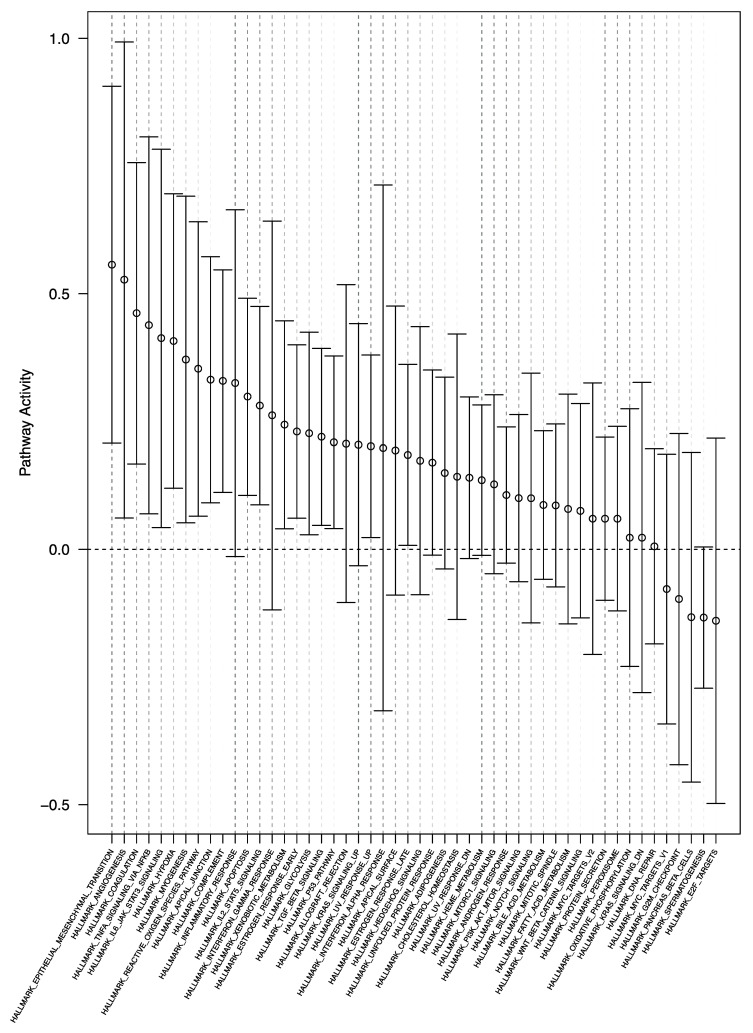
Pathway enrichment plot of the *withinSVZ-*samples vs. *noSVZcontact-*samples analysis. Gene set enrichment analysis of the *withinSVZ-*samples vs. *noSVZcontact*-samples showed differential enrichment of 27 gene sets (*p* < 0.05, FDR < 0.25), which are shown on the *x*-axis. The relative gene set activity (log fold change) is displayed on the *y*-axis. The 95% confidence intervals are represented by the horizontal bars. Most gene sets were enriched in the *withinSVZ*-samples group, as shown by their positive log fold changes.

Gene set enrichment analysis showed differential expression of 27 gene sets (*p* < 0.05, FDR < 0.25, Supplementary Table S3, Figure 4). Again, we found relatively increased enrichment of gene sets involved in (epithelial-)mesenchymal transition, angiogenesis, and NF-κB and JAK/STAT3 signaling in the *withinSVZ*-samples.

### 3.3. No Difference in Oncogenic Signaling between Glioblastoma Samples from Outside the SVZ and Tumors without SVZ Contact: Intertumoral Analysis

In this analysis, 64 tumor samples (36 *outsideSVZ*-samples and 28 *noSVZcontact*-samples) were included, from 24 patients (Figure 1).

No significantly differentially expressed genes between the *outsideSVZ*-samples and *noSVZcontact*-samples were found (adjusted *p*-values > 0.50 for all genes). The results for the 50 genes with the lowest adjusted *p*-values are shown in a heatmap (Supplementary Figure S5). No differential enrichment of gene sets (with an FDR less than 0.25) were found.

## 4. Discussion

With intra- and intertumoral gene expression analyses, we show that gene sets associated with mesenchymal transition are relatively enriched in glioblastoma tissue in the SVZ region, as compared to tumor tissue outside the SVZ region. To our knowledge, this is the first study in which gene expression in glioblastomas contacting the SVZ is regionally explored to this end so far. Our findings suggest that the SVZ microenvironment could influence oncogenic signaling in glioblastoma.

The differential enrichment of gene sets was more pronounced in the comparison of *withinSVZ*-samples versus *noSVZcontact*-samples, than in the comparison of different samples from the SVZ-contacting tumors (*withinSVZ* versus *outsideSVZ*), which could reflect a greater degree of similarity in the samples in the latter comparison. The comparison of *outsideSVZ*-samples from the SVZ-contacting tumors with *noSVZcontact*-samples from glioblastomas without SVZ contact showed no significantly different gene or pathway expression. This is in line with the hypothesis that the SVZ microenvironment could influence region-specific gene expression in glioblastoma.

Few studies have focused on the interaction of the SVZ microenvironment and glioblastoma (stem) cells (GSCs) [18,19,20]. This is of particular interest, as the SVZ niche is believed to serve as a GSC reservoir which contributes to resistance to therapy. It is proposed that the microenvironment in the SVZ closely interacts with GSCs in order to establish this protective niche [18,19,21]. One study showed that GSCs in the SVZ appear to have an enhanced mesenchymal signature compared with their counterparts from the tumor [19]. These mesenchymal features, including a higher expression level of N-cadherin and vimentin, were shown to be upregulated by SVZ-released CXCL12. Moreover, inhibition of the CXCL12/CXCR4 signaling axis with AMD3100 (a CXCL12/CXCR4 antagonist) weakened the tumor’s mesenchymal signature in the SVZ and increased the tumor’s sensitivity to radiotherapy [19]. This correlation of mesenchymal activation in glioblastoma and resistance to radiotherapy (and chemotherapy) has been reported in other studies as well [22]. Another study found a similar predominance of the mesenchymal subtype in glioblastoma samples from the SVZ as compared to SVZ distant samples from the same tumor [20]. Moreover, in this study, isolated GSCs from the SVZ and GSCs from other tumor mass of the same glioblastoma showed different patterns of response to therapies [20]. In addition, neural-precursor cells can secrete factors such as PTN and ROCK that were shown to drive glioblastoma invasion to the SVZ. In our analysis, for instance, ROCK1 is included in, among others, the HALLMARK_APOPTOSIS pathway, which is enriched in *withinSVZ* tissue samples [23].

In this light, our results provide further evidence to this SVZ region-specific mesenchymal transition in glioblastoma. However, a deeper insight into the complex interaction between glioblastoma cells (and GSCs in particular) and the SVZ micro-environment is needed to unravel the mechanism behind this mesenchymal shift.

In addition to the (epithelial-)mesenchymal transition signaling pathway, several other gene sets linked to aggressive growth of glioblastoma were relatively upregulated in the *withinSVZ*-samples group, including TNF-α-mediated NF-κB activation, IL-6 induced STAT3 activation, TGF-β signaling, p53 signaling, KRas signaling, genes upregulated by reactive oxygen species, angiogenesis, hypoxia, coagulation, complement activation and inflammatory response. Activation of and interaction between several of these pathways have been linked to induction of (epithelial-)mesenchymal transition in glioblastoma as well [22,24,25,26,27,28,29,30]. For example, the mesenchymal subtype of glioblastoma is characterized by increased levels of NF-κB signaling components and moreover, glioma sphere cultures of the proneural subtype can transform to a mesenchymal state under TNF-α-mediated NF-κB activation [22]. Additionally, it has been demonstrated that STAT3 activation increases when initial proneural tumors experience a mesenchymal shift upon recurrence [31,32]. Furthermore, STAT3 was shown to be highly expressed in glioblastoma stem cells (GSCs), which are tumor cells with self-renewing properties that contribute to tumor initiation and therapeutic resistance [33]. Finally, hypoxia and reactive oxygen species could also potentially induce mesenchymal transition in glioblastoma [27,34].

The only significantly differentially expressed gene (DCAF4) was observed in the *withinSVZ*-samples vs. *noSVZcontact*-samples analysis. DCAF4 (DDB1 and CUL4-associated factor 4) encodes a WD (Trp-Asp) repeat-containing protein that interacts with the CUL4-DDB1 E3 ligase macromolecular complex. The CUL4-DDB1 ubiquitin ligase is involved in cell proliferation, survival, DNA repair and genomic integrity [35]. CUL4A demonstrated the potential to promote (epithelial-)mesenchymal transition through the activation of ZEB1 in breast cancer cells [36]. One study showed that overexpression of DCAF4L2, a paralog of DCAF4, could support (epithelial-)mesenchymal transition in colorectal cancer cells through activation of the NF-κB pathway [37], and genetic variations in DCAF4 have been previously associated with leukocyte telomere length, keloid formation and lung cancer risk [38,39,40]. However, as we found a relative downregulation of DCAF4 in nearly all *withinSVZ*-samples, the relevance of this observation compared to increased expression of gene sets involved in (epithelial-) mesenchymal transition in tumor samples from the SVZ region remains unclear.

Our study has some technical limitations that should be considered. We relied on the availability of data from the comprehensive Ivy Glioblastoma Atlas. RNA sequencing data were not available for all tissue regions and spatial orientation of the tumor tissues was not clear in some cases. As a consequence, only a limited number of patients could be included in our analyses. Especially for the samples within the SVZ region, the number of available tumor samples was limited (nine samples from seven patients). Given the small number of patients, we were unable to study our findings in relevant subgroups, such as different glioblastoma subtypes or varying subventricular subregions.

Although the gene set expression results of our separate subanalyses are in line with each other, gene set analyses in relatively small-sized cohorts should be regarded as exploratory, and must be validated in other studies prior to drawing any conclusions. Future studies could elucidate the region-specific molecular profiles in SVZ-regions with macroscopic tumor involvement as well as normal-appearing SVZ, through prospective collection of relevant tissue and detailed analysis with single-cell RNA sequencing.

## 5. Conclusions

Glioblastoma tissues within the SVZ region show increased expression of gene sets involved in (epithelial-)mesenchymal transition, angiogenesis, and NF-κB and JAK/STAT3 signaling, compared to tumor tissues from non-SVZ-regions, either from the same SVZ-contacting tumors or from glioblastomas that do not contact the SVZ. Our results suggest that the SVZ microenvironment could influence regional gene expression in glioblastoma, to induce (epithelial-)mesenchymal transition and possibly a more invasive glioblastoma phenotype.

## Figures and Tables

**Table 1:** Baseline characteristics.

	SVZ (n = 16)	Non-SVZ (n = 10)	*p*-Value
**Gender** n (%)			0.68
Male	7 (43.8%)	3 (30%)	
Female	9 (56.3%)	7 (70%)	
**Age** mean (SD)	62.1 (6.3)	60.2 (8.4)	0.51
**Initial KPS** median (IQR)	90 (28)	90 (20)	0.76
**Extent of resection** n (%)			0.35
Total	11 (68.8%)	9 (90%)	
Subtotal	5 (31.3%)	1 (10%)	
**Chemo and/or radiotherapy** n (%)			0.29
Both	14 (87.5%)	9 (90%)	
Only chemotherapy	0	1 (10%)	
Only radiotherapy	0	0	
None	2 (12.5%)	0	
**PTEN** n (%)			0.52
Loss	11 (68.8%)	7 (70%)	
Normal	2 (12.5%)	0	
Gain	1 (6.2%)	1 (10%)	
Missing	2 (12.5%)	2 (20%)	
**MGMT Methylation** n (%)			0.4
Yes	7 (43.8%)	2 (20%)	
No	9 (56.3%)	8 (80%)	
**EGFR Amplification** n (%)			1
Yes	8 (50.0%)	4 (40%)	
No	6 (37.5%)	4 (40%)	
Missing	2 (12.5%)	2 (20%)	
**EGFR vIII** n (%)			0.62
Yes	3 (18.8%)	3 (30%)	
No	11 (68.8%)	5 (50%)	
Missing	2 (12.5%)	2 (20%)	
**Survival** in days, median (IQR)	442 (119)	544 (137)	0.23

Abbreviations: SVZ: subventricular zone, SD: standard deviation, KPS: Karnofsky Performance Score, IQR: interquartile range, PTEN: phosphatase and tensin homolog, MGMT: O6-methylguanine–DNA methyltransferase, EGFR: epidermal growth factor receptor.

## Data Availability

Publicly available datasets were analyzed in this study. This data can be found here: https://glioblastoma.alleninstitute.org/.

## Supplementary Materials

The following are available online at https://www.mdpi.com/article/10.3390/cancers13153764/s1, Figure S1: Kaplan meier survival analysis, Figure S2: Top 50 most differentially expressed genes, *withinSVZ* samples vs. *outsideSVZ* samples, Figure S3: DCAF4 expression plot, Figure S4: Top 50 most differentially expressed genes, *withinSVZ* samples vs. *noSVZcontact* samples, Figure S5: Top 50 most differentially expressed genes, *outsideSVZ* samples vs. *noSVZcontact* samples, Table S1: Verhaak subtype(s) in SVZ-contacting glioblastomas and glioblastomas without SVZ contact, Table S2: Hallmark genesets in within SVZ-contacting glioblastoma (*withinSVZ*-samples vs. *outsideSVZ*-samples) analysis with FDR < 0.25 sorted by *p*-value. Table S3: Hallmark genesets in *withinSVZ*-samples vs. *noSVZcontact*-samples analysis with FDR < 0.25 sorted by *p*-value.

## References

[1] Stupp R., Hegi M.E., Mason W.P., van den Bent M.J., Taphoorn M.J.B., Janzer R.C., Ludwin S.K., Allgeier A., Fisher B., Belanger K. (2009). Effects of radiotherapy with concomitant and adjuvant temozolomide versus radiotherapy alone on survival in glioblastoma in a randomised phase III study: 5-year analysis of the EORTC-NCIC trial. Lancet Oncol..

[2] Sanai N., Tramontin A.D., Quinones-Hinojosa A., Barbaro N.M., Gupta N., Kunwar S., Lawton M.T., McDermott M.W., Parsa A.T., García-Verdugo J.M. (2004). Unique astrocyte ribbon in adult human brain contains neural stem cells but lacks chain migration. Nature.

[3] Llaguno S.A., Chen J., Kwon C.H., Jackson E.L., Li Y., Burns D.K., Alvarez-Buylla A., Parada L.F. (2009). Malignant astrocytomas originate from neural stem/progenitor cells in a somatic tumor suppressor mouse model. Cancer Cell.

[4] Lee J.H., Lee J.E., Kahng J.Y., Kim S.H., Park J.S., Yoon S.J., Um J., Kim W.K., Lee J.K., Park J. (2018). Human glioblastoma arises from subventricular zone cells with low-level driver mutations. Nature.

[5] Berendsen S., van Bodegraven E., Seute T., Spliet W.G.M., Geurts M., Hendrikse J., Schoysman L., Huiszoon W.B., Varkila M., Rouss S. (2019). Adverse prognosis of glioblastoma contacting the subventricular zone: Biological correlates. PLoS ONE.

[6] Mistry A.M., Wooten D.J., Davis L.T., Mobley B.C., Quaranta V., Ihrie R.A. (2019). Ventricular-Subventricular Zone Contact by Glioblastoma is Not Associated with Molecular Signatures in Bulk Tumor Data. Sci. Rep..

[7] Jungk C., Mock A., Exner J., Geisenberger C., Warta R., Capper D., Abdollahi A., Friauf S., Lahrmann B., Grabe N. (2016). Spatial transcriptome analysis reveals Notch pathway-associated prognostic markers in IDH1 wild-type glioblastoma involving the subventricular zone. BMC Med..

[8] Denicolaï E., Tabouret E., Colin C., Metellus P., Nanni I., Boucard C., Tchoghandjian A., Meyronet D., Baeza-Kallee N., Chinot O. (2016). Molecular heterogeneity of glioblastomas; does location matter?. Oncotarget.

[9] Jamshidi N., Diehn M., Bredel M., Kuo M.D. (2014). Illuminating radiogenomic characteristics of glioblastoma multiforme through integration of MR imaging, messenger RNA expression, and DNA copy number variation. Radiology.

[10] Kappadakunnel M., Eskin A., Dong J., Nelson S.F., Mischel P.S., Liau L.M., Ngheimphu A.L., Cloughesy T.F., Goldin J., Pope W.B. (2010). Stem cell associated gene expression in glioblastoma multiforme: Relationship to survival and the subventricular zone. J. Neurooncol..

[11] Han S., Li X., Qiu B., Jiang T., Wu A. (2015). Can lateral ventricle contact predict the ontogeny and prognosis of glioblastoma?. J. Neurooncol..

[12] Gollapalli K., Ghantasala S., Kumar S., Srivastava R., Rapole S., Moiyadi S.E., Srivastava S. (2017). Subventricular zone involvement in Glioblastoma—A proteomic evaluation and clinicoradiological correlation. Sci. Rep..

[13] Verhaak R.G.W., Hoadley K.A., Purdom E., Wang V., Qi Y., Wilkerson M.D., Miller R., Ding L., Golub T., Mesirov J.P. (2010). Integrated genomic analysis identifies clinically relevant subtypes of glioblastoma characterized by abnormalities in PDGFRA, IDH1, EGFR, and NF1. Cancer Cell.

[14] Sottoriva A., Spiteri I., Piccirillo S.G., Peter Collins V., Marioni J.C., Curtis C., Watts C., Tavaré S. (2013). Intratumor heterogeneity in human glioblastoma reflects cancer evolutionary dynamics. Proc. Natl. Acad. Sci. USA.

[15] Bonavia R., Inda M., Cavenee W.K., Furnari F.B. (2011). Heterogeneity maintenance in glioblastoma: A social network. Cancer Res..

[16] Puchalski R.B., Shah N., Miller J., Dalley R., Nomura S.R., Yoon J., Smith K.A., Lankerovich M., Bertagnolli D., Brickley K. (2018). An anatomic transcriptional atlas of human glioblastoma. Science.

[17] Liberzon A., Birger C., Thorvaldsdóttir H., Ghandi M., Mesirov J.P., Tamayo P. (2015). The Molecular Signatures Database (MSigDB) hallmark gene set collection. Cell Syst..

[18] Goffart N., Kroonen J., Di Valentin E., Dedobbeleer M., Denne A., Martinive P., Rogister B. (2015). Adult mouse subventricular zones stimulate glioblastoma stem cells specific invasion through CXCL12/CXCR4 signaling. Neuro Oncol..

[19] Goffart N., Lombard A., Lallemand F., Kroonen J., Nassen J., Di Valentin E., Berendsen S., Dedobbeleer M., Willems E., Robe P.A. (2017). CXCL12 mediates glioblastoma resistance to radiotherapy in the subventricular zone. Neuro Oncol..

[20] Piccirillo S.G.M., Spiteri I., Sottoriva A., Touloumis A., Ber S., Price S.J., Heywood R., Francis N., Howarth K.D., Collins V.P. (2015). Contributions to drug resistance in glioblastoma derived from malignant cells in the sub-ependymal zone. Cancer Res..

[21] Kroonen J., Nassen J., Boulanger Y.G., Provenzano F., Capraro V., Bours V., Martin D., Deprez M., Robe P.A., Rogister B. (2011). Human glioblastoma-initiating cells invade specifically the subventricular zones and olfactory bulbs of mice after striatal injection. Int. J. Cancer.

[22] Bhat K.P.L., Balasubramaniyan V., Vaillant B., Ezhilarasan R., Hummelink K., Hollingsworth F., Wani K., Heathcock L., James J.D., Goodman L.D. (2013). Mesenchymal differentiation mediated by NF-kappaB promotes radiation resistance in glioblastoma. Cancer Cell.

[23] Qin E.Y., Cooper D.D., Abbott K.L., Lennon J., Nagaraja S., Mackay A., Jones C., Vogel H., Jackson P.K., Monje M. (2017). Neural precursor-derived pleiotrophin mediates subventricular zone invasion by glioma. Cell.

[24] Conroy S., Kruyt F.A.E., Wagemakers M., Bhat K.P.L., den Dunnen W.F.A. (2018). IL-8 associates with a pro-angiogenic and mesenchymal subtype in glioblastoma. Oncotarget.

[25] Xie T.X., Xia Z., Zhang N., Gong W., Huang S. (2010). Constitutive NF-kappaB activity regulates the expression of VEGF and IL-8 and tumor angiogenesis of human glioblastoma. Oncol. Rep..

[26] Talasila K.M., Røsland G.V., Hagland H.R., Eskilsson E., Flones I.H., Fritah S., Azuaje F., Atai N., Harter P.N., Mittelbronn M. (2017). The angiogenic switch leads to a metabolic shift in human glioblastoma. Neuro Oncol..

[27] Joseph J.V., Conroy S., Pavlov K., Sontakke P., Tomar T., Eggens-Meijer E., Balasubramaniyan V., Wagemakers M., den Dunnen W.F.A., Kruyt F.A.E. (2015). Hypoxia enhances migration and invasion in glioblastoma by promoting a mesenchymal shift mediated by the HIF1alpha-ZEB1 axis. Cancer Lett..

[28] Magnus N., Gerges N., Jabado N., Rak J. (2013). Coagulation-related gene expression profile in glioblastoma is defined by molecular disease subtype. J. Thromb. Haemost..

[29] Carro M.S., Lim W.K., Alvarez M.J., Bollo R.J., Zhao X., Snyder E.Y., Sulman E.P., Anne S.L., Doetsch F., Colman H. (2010). The transcriptional network for mesenchymal transformation of brain tumours. Nature.

[30] Cooper L.A.D., Gutman D.A., Chisolm C., Appin C., Kong J., Rong Y., Kurc T., van Meir E., Saltz J.H., Moreno C.S. (2012). The tumor microenvironment strongly impacts master transcriptional regulators and gene expression class of glioblastoma. Am. J. Pathol..

[31] Phillips H.S., Kharbanda S., Chen R., Forrest W.F., Soriano R.H., Wu T.D., Misra A., Nigro J.M., Colman H., Soroceanu L. (2006). Molecular subclasses of high-grade glioma predict prognosis, delineate a pattern of disease progression, and resemble stages in neurogenesis. Cancer Cell.

[32] Halliday J., Helmy K., Pattwell S.S., Pitter K.L., LaPlant Q., Ozawa T., Holland E.C. (2014). In vivo radiation response of proneural glioma characterized by protective p53 transcriptional program and proneural-mesenchymal shift. Proc. Natl. Acad. Sci. USA.

[33] Sherry M.M., Reeves A., Wu J.K., Cochran B.H. (2009). STAT3 is required for proliferation and maintenance of multipotency in glioblastoma stem cells. Stem Cells.

[34] Singer E., Judkins J., Salomonis N., Matlaf L., Soteropoulos P., McAllister S., Soroceanu L. (2015). Reactive oxygen species-mediated therapeutic response and resistance in glioblastoma. Cell Death Dis..

[35] Lee J., Zhou P. (2007). DCAFs, the missing link of the CUL4-DDB1 ubiquitin ligase. Mol. Cell.

[36] Wang Y., Wen M., Kwon Y., Xu Y., Liu Y., Zhang P., He X., Wang Q., Huang Y., Jen K. (2014). CUL4A induces epithelial-mesenchymal transition and promotes cancer metastasis by regulating ZEB1 expression. Cancer Res..

[37] Wang H., Chen Y., Han J., Meng Q., Xi Q., Wu G., Zhang B. (2016). DCAF4L2 promotes colorectal cancer invasion and metastasis via mediating degradation of NFkappab negative regulator PPM1B. Am. J. Transl. Res..

[38] Mangino M., Christiansen L., Stone R., Hunt S.C., Horvath K., Eisenberg D.T.A., Kimura M., Petersen I., Kark J.D., Herbig U. (2015). DCAF4, a novel gene associated with leucocyte telomere length. J. Med. Genet.

[39] Hellwege J.N., Russell S.B., Williams S.M., Edwards T.L., Velez Edwards D.R. (2018). Gene-based evaluation of low-frequency variation and genetically-predicted gene expression impacting risk of keloid formation. Ann. Hum. Genet.

[40] Liu H., Liu Z., Wang Y., Stinchcombe T.E., Owzar K., Han Y., Hung R.J., Brhane Y., McLaughlin J., Brennan P. (2017). Functional variants in DCAF4 associated with lung cancer risk in European populations. Carcinogenesis.

