# Supplementary material for: Glioblastomas within the Subventricular Zone Are Region-Specific Enriched for Mesenchymal Transition Markers: An Intratumoral Gene Expression Analysis
Source: Cancers (Basel). 2021 Jul 27;13(15):3764. doi: 10.3390/cancers13153764 (PMC8345101; doi:10.3390/cancers13153764)
Supplement: Supplementary file 1 [file cancers-13-03764-s001.zip › Supplementary table1.pdf]

Supplementary Table S1: Verhaak subtype(s) in SVZ-contacting glioblastomas and glioblastomas without SVZ contact

**SVZ-contacting glioblastomas (n=16)**	**Glioblastomas without SVZ contact (n=10)**
W1-1-2: Classical	W3-1-1: Classical, Mesenchymal
W2-1-1: Classical, Neural	W5-1-1: Classical, Neural
W4-1-1: Mesenchymal, Neural	W8-1-1: Classical, Mesenchymal
W6-1-1: Mesenchymal	W9-1-1: Proneural
W7-1-1: Mesenchymal	W16-1-1: Neural, Proneural
W11-1-1: Classical, Mesenchymal	W19-1-1: Proneural
W12-1-1: Classical	W22-1-1: Classical, Neural
W13-1-1: Mesenchymal	W26-1-1: Neural
W21-1-1: Proneural	W36-1-1: Mesenchymal
W28-1-1: Mesenchymal, Neural	W43-1-1: Mesenchymal, Neural
W29-1-1: Classical, Neural	
W32-1-1: Proneural	
W38-1-1: Proneural	
W39-1-1: Classical	
W40-1-1: Neural	
W42-1-1: Neural, Proneural	

The Verhaak classification is a robust gene expression-based molecular classification of glioblastoma into Proneural, Neural, Classical, and Mesenchymal subtypes. WX-X-X stands for the patient number in the Ivy Glioblastoma Atlas.
