# Supplementary material for: Glioblastomas within the Subventricular Zone Are Region-Specific Enriched for Mesenchymal Transition Markers: An Intratumoral Gene Expression Analysis
Source: Cancers (Basel). 2021 Jul 27;13(15):3764. doi: 10.3390/cancers13153764 (PMC8345101; doi:10.3390/cancers13153764)
Supplement: Supplementary file 1 [file cancers-13-03764-s001.zip › Supplementarytable2.pdf]

Supplementary Table S2. Hallmark genesets in within SVZ-contacting glioblastoma (withinSVZ-samples vs outsideSVZ-samples) analysis with FDR<0.25 sorted by p-value. Most gene sets are upregulated in the withinSVZ-samples group, as shown by their positive log fold changes (logFC).

**Pathway name**	**Log Fold Change**	**p-value**	**FDR**
EPITHELIAL_MESENCHYMAL_TRANSITION	0.41	0.003	0.13
ANGIOGENESIS	0.40	0.006	0.13
APOPTOSIS	0.21	0.01	0.13
IL6_JAK_STAT3_SIGNALING	0.37	0.01	0.13
SPERMATOGENESIS	-0.18	0.01	0.13
COMPLEMENT	0.22	0.02	0.13
UV_RESPONSE_DN	0.12	0.02	0.13
ALLOGRAFT_REJECTION	0.22	0.03	0.13
COAGULATION	0.27	0.03	0.13
IL2_STAT5_SIGNALING	0.18	0.03	0.13
ESTROGEN_RESPONSE_EARLY	0.14	0.04	0.15
INFLAMMATORY_RESPONSE	0.27	0.04	0.15
INTERFERON_GAMMA_RESPONSE	0.26	0.04	0.15
P53_PATHWAY	0.14	0.04	0.15
TGF_BETA_SIGNALING	0.14	0.04	0.15
APICAL_JUNCTION	0.17	0.05	0.16
TNFA_SIGNALING_VIA_NFKB	0.35	0.06	0.16
ESTROGEN_RESPONSE_LATE	0.11	0.06	0.17
HYPOXIA	0.27	0.07	0.17
KRAS_SIGNALING_UP	0.15	0.07	0.17
MYOGENESIS	0.20	0.07	0.17
XENOBIOTIC_METABOLISM	0.13	0.09	0.21
REACTIVE_OXIGEN_SPECIES_PATHWAY	0.17	0.11	0.24
APICAL_SURFACE	0.12	0.12	0.24
