# Supplementary material for: Glioblastomas within the Subventricular Zone Are Region-Specific Enriched for Mesenchymal Transition Markers: An Intratumoral Gene Expression Analysis
Source: Cancers (Basel). 2021 Jul 27;13(15):3764. doi: 10.3390/cancers13153764 (PMC8345101; doi:10.3390/cancers13153764)
Supplement: Supplementary file 1 [file cancers-13-03764-s001.zip › Supplementarytable3.pdf]

Supplementary Table S3. Hallmark genesets in withinSVZ-samples vs noSVZcontact-samples analysis with FDR<0.25 sorted by p-value.
Most gene sets are upregulated in the withinSVZ-samples group, as shown by their positive log fold changes (logFC).

Pathway name	Log Fold Change	p-value	FDR
EPITHELIAL_MESENCHYMAL_TRANSITION	0.56	0.002	0.04
COAGULATION	0.46	0.002	0.04
APOPTOSIS	0.30	0.002	0.04
COMPLEMENT	0.33	0.003	0.04
IL2_STAT5_SIGNALING	0.28	0.005	0.05
HYPOXIA	0.41	0.006	0.05
APICAL_JUNCTION	0.33	0.007	0.05
ESTROGEN_RESPONSE_EARLY	0.23	0.008	0.05
TGF_BETA_SIGNALING	0.22	0.01	0.07
TNFA_SIGNALING_VIA_NFKB	0.44	0.02	0.08
MYOGENESIS	0.37	0.02	0.08
REACTIVE_OXIGEN_SPECIES_PATHWAY	0.35	0.02	0.08
XENOBIOTIC_METABOLISM	0.24	0.02	0.08
P53_PATHWAY	0.21	0.02	0.08
ANGIOGENESIS	0.53	0.03	0.08
IL6_JAK_STAT3_SIGNALING	0.41	0.03	0.08
GLYCOLYSIS	0.23	0.03	0.08
UV_RESPONSE_UP	0.20	0.03	0.08
ESTROGEN_RESPONSE_LATE	0.18	0.04	0.11
INFLAMMATORY_RESPONSE	0.33	0.06	0.14
SPERMATOGENESIS	-0.13	0.06	0.14
UNFOLDED_PROTEIN_RESPONSE	0.17	0.07	0.15
HEME_METABOLISM	0.14	0.07	0.16
UV_RESPONSE_DN	0.14	0.08	0.17
KRAS_SIGNALING_UP	0.20	0.09	0.18
ADIPOGENESIS	0.15	0.12	0.22
ANDROGEN_RESPONSE	0.11	0.12	0.22
