# Supplementary figures and images for: Glioblastomas within the Subventricular Zone Are Region-Specific Enriched for Mesenchymal Transition Markers: An Intratumoral Gene Expression Analysis
Source: Cancers (Basel). 2021 Jul 27;13(15):3764. doi: 10.3390/cancers13153764 (PMC8345101; doi:10.3390/cancers13153764)

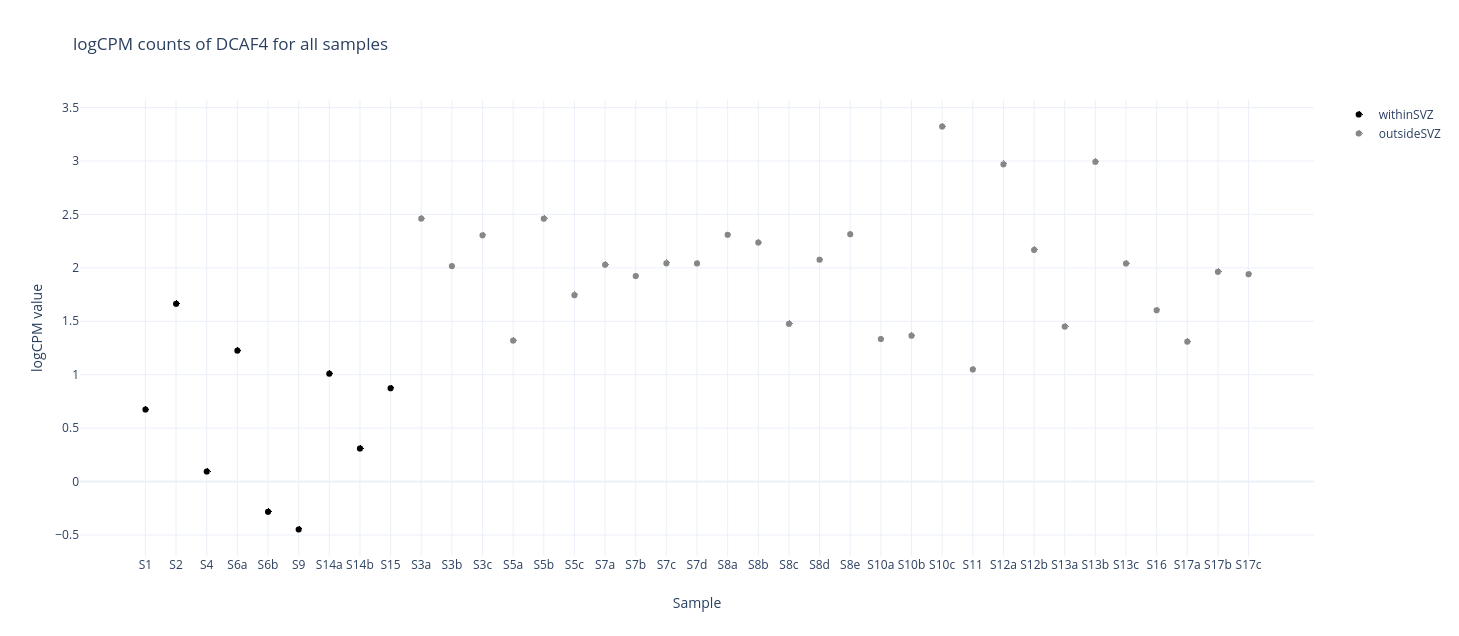

Supplement: Supplementary file 1 [file cancers-13-03764-s001.zip › Supplementaryfigure3.tif]
